# Pathophysiology of Cerebral Hyperperfusion in Term Neonates With Hypoxic-Ischemic Encephalopathy: A Systematic Review for Future Research

**DOI:** 10.3389/fped.2021.631258

**Published:** 2021-02-02

**Authors:** Dianne G. Kleuskens, Filipe Gonçalves Costa, Kim V. Annink, Agnes van den Hoogen, Thomas Alderliesten, Floris Groenendaal, Manon J. N. Benders, Jeroen Dudink

**Affiliations:** Department of Neonatology, Wilhelmina Children's Hospital, University Medical Center Utrecht, Utrecht University, Utrecht, Netherlands

**Keywords:** cerebral hyperperfusion, cerebral vasodilation, hyperemia, hypoxic-ischemic encephalopathy (HIE), neonatal encephalopathy, perinatal hypoxia-ischemia

## Abstract

Worldwide neonatal hypoxic-ischemic encephalopathy (HIE) is a common cause of mortality and neurologic disability, despite the implementation of therapeutic hypothermia treatment. Advances toward new neuroprotective interventions have been limited by incomplete knowledge about secondary injurious processes such as cerebral hyperperfusion commonly observed during the first 1–5 days after asphyxia. Cerebral hyperperfusion is correlated with adverse neurodevelopmental outcome and it is a process that remains poorly understood. In order to provide an overview of the existing knowledge on the pathophysiology and highlight the gaps in current understanding of cerebral hyperperfusion in term animals and neonates with HIE, we performed a systematic research. We included papers scoping for study design, population, number of participants, study technique and relevant findings. Methodological quality was assessed using the checklist for cohort studies from The Joanna Briggs Institute. Out of 2,690 results, 34 studies were included in the final review—all prospective cohort studies. There were 14 studies of high, 17 moderate and 3 of low methodological quality. Data from the literature were analyzed in two main subjects: (1) Hemodynamic Changes subdivided into macro- and microscopic hemodynamic changes, and (2) Endogenous Pathways which was subdivided into N-methyl-D-aspartate/Mitogen activated protein kinase (NDMA/MAPK), Nitric Oxide (NO), prostanoids and other endogenous studies. Cerebral hyperperfusion in term neonates with HIE was found to be present 10–30 min after the hypoxic-ischemic event and was still present around day 10 and up to 1 month after birth. Cerebral hyperperfusion was also characterized by angiogenesis and cerebral vasodilation. Additionally, cerebral vasodilation was mediated by endogenous pathways such as MAPK through urokinase Plasminogen Activator (uPA), by neuronal NO synthase following NMDA and by prostanoid synthesis. Future research should elucidate the precise role of NMDA, MAPK and prostanoids in cerebral hyperperfusion. Moreover, research should focus on possible interventions and the effect of hypothermia on hyperperfusion. These findings should be taken into account simultaneously with brain imagining techniques, becoming a valuable asset in assessing the impact in neurodevelopmental outcome.

## Highlights

- What is already known? Cerebral hyperperfusion in term neonates with hypoxic-ischemic encephalopathy is often seen after the acute phase, occurring 6–15 h after the HI-event. Cerebral hyperperfusion is correlated with adverse neurodevelopmental outcome.
- What this review adds? Cerebral hyperperfusion is present 10–30 minutes after the hypoxic-ischemic event and persisted around day 10 and up to 1 month after birth. Cerebral hyperperfusion may be characterized by angiogenesis and cerebral vasodilation. A rise in prostanoids may contribute to vasodilatation trough MAPK. Another finding is that NMDA induces vasodilatation and the cerebroprotective effects of hypothermia therapy are not mediated by NMDA.
- What is still unknown? Future research needs to be developed about the precise role of NMDA, MAPK, prostanoids in cerebral vasodilation, and possible interventions.

## Introduction

Hypoxic-ischemic encephalopathy (HIE) is one of the main causes of neonatal death and developmental psychomotor disorders in the pediatric population. The incidence of HIE is ~1–8 per 1,000 live births in technically advanced countries and is up to 26 per 1,000 live births in less developed countries (1). Despite the widespread use of therapeutic hypothermia, a large proportion of infants will suffer from neurodevelopmental impairments, especially in the case of severe HIE (2). Therefore, new synergistic therapies need to be developed.

**Figure 1:**
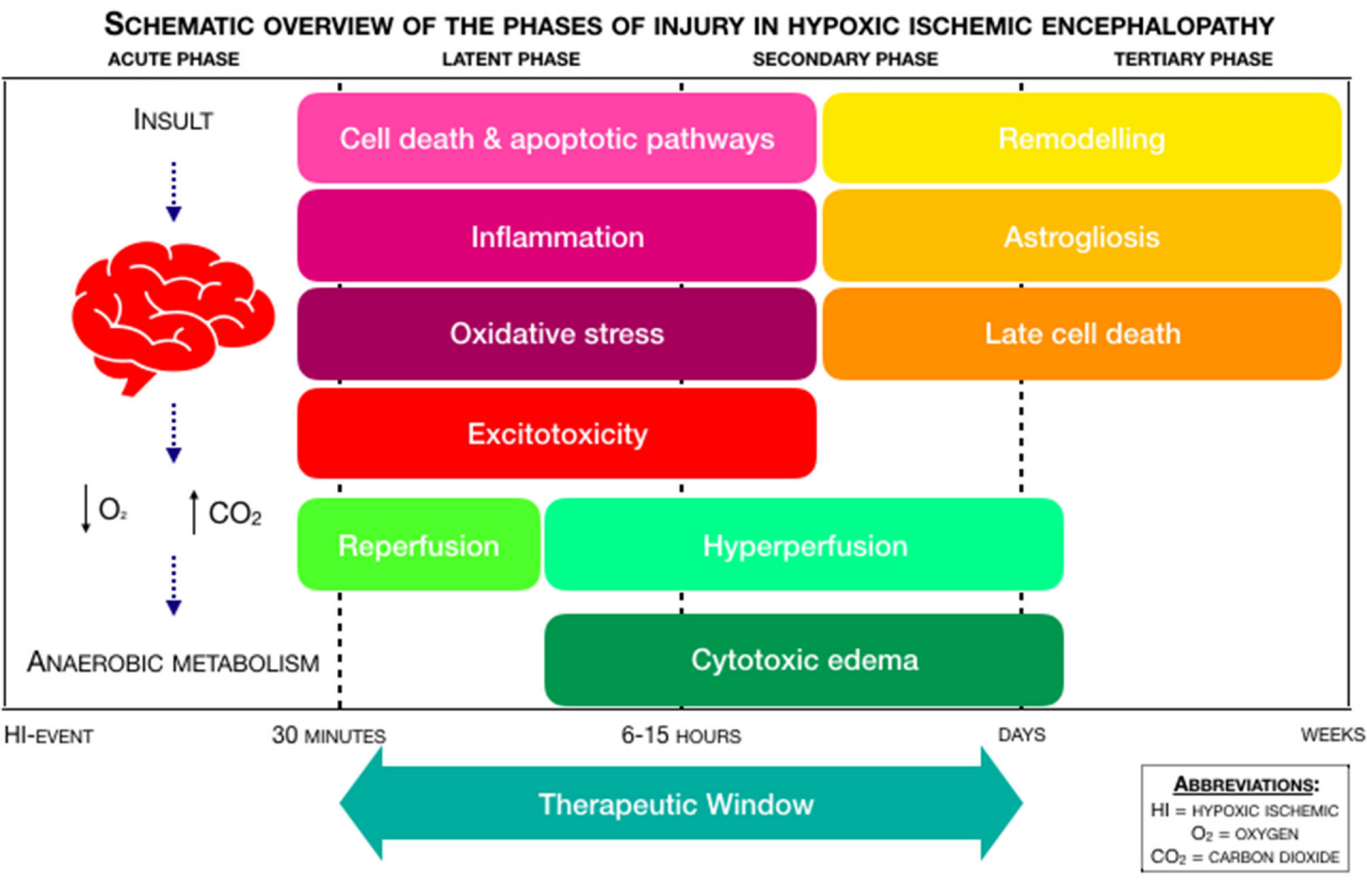
Schematic overview of the phases of injury in Hypoxic-Ischemic Encephalopathy (HIE), adapted from Douglas-Escobar et al. (4) and Hassell et al. (5).

For clinicians, it is important to understand the pathophysiology of injury after the HI-event, due to its relevance in developing novel therapies and because neuroimaging has shown that subsequent brain injury evolves over the course of days, if not weeks (3). In the process of neonatal encephalopathy, a hypoxic-ischemic event, with intermitting anoxia or acute hypoxia, leads to a decreased cerebral perfusion, if the event occurs for long enough. This decreased perfusion sets a time-related pattern of injury pathways in motion, which is divided into distinct phases (4, 5). Figure 1 provides a schematic overview of the phases of injury in HIE. After the hypoxic-ischemic event, there is the acute phase of injury, which is followed by a latent phase of injury. After ~6–15 h, there is a secondary phase. A tertiary phase occurs weeks after the hypoxic-ischemic event. More extensive:

- The **acute phase**, also known as “primary energy failure,” is characterized by anaerobic metabolism, oxidative stress, neuronal cell death, and excitotoxicity. In the process of excitotoxicity, a surplus in the amount of the excitatory amino acid glutamate leads to overstimulation of the 2-(aminomethyl)phenylacetic acid (AMPA), kainite (KA), and N-methyl-D-aspartate (NMDA) receptors. Overstimulation of AMPA and KA causes an influx of sodium (Na^+^) and chloride (Cl^−^), which leads to an increased cellular osmolality. Overstimulation of NMDA triggers the influx of calcium (Ca^2+^), which leads to apoptosis and necrosis (4). The mitogen activated protein kinase (MAPK) signaling pathway is overactivated after the hypoxic-ischemic event, leading to neuronal apoptosis. Prostanoids also play a role in the hypoxic-ischemic event: stimulating prostanoid-receptors may be neuroprotective and prevent neuronal cerebellar injury (4). Depending on the duration of decreased perfusion and the presence or absence of medical intervention, a partial recovery with reperfusion occurs from 30 to 60 min after the hypoxic-ischemic event (6).

- The **latent phase** of injury follows after the acute phase, which may last from 1 to 6 h. This latent phase is characterized by neuroinflammation and the continuation of activated apoptotic cascades. In neonates with moderate to severe HIE, the latent phase is followed up by the secondary phase, also known as “secondary energy failure” (4).

- The **secondary phase** occurs within ~6–15 h and is characterized by cytotoxic edema, excitotoxicity and cerebral hyperperfusion (7).

- The **tertiary phase** occurs in the weeks or months following primary energy failure. It involves remodeling of the injured brain, astrogliosis, and late cell death (8).

In this review, we focused on pathophysiology of cerebral hyperperfusion, often seen in the secondary phase of HI-injury, and not on the adverse outcome itself. Cranial ultrasound (cUS) and brain Magnetic Resonance Imaging (MRI) are the most used imaging techniques in neonates with HIE. Cerebral hyperperfusion seen on Arterial Spin Labeling (ASL) MRI is correlated with an adverse neurodevelopmental outcome (2, 9). To understand cerebral hyperperfusion, it is essential to elucidate when this phenomenon takes place and what the underlying mechanisms leading to hyperperfusion are. Understanding the pathophysiological mechanisms behind these observations will provide an insight into how cerebral hyperperfusion is associated with (adverse) outcome.

Given that this topic is prone to rapid changes in what is known to the scientific community, the review intents to provide a summary of the methods, models and studies already existing. With this paper we hope to create a platform that allows researchers to come up with novel ideas, using this review as a gateway for new research. This study aims to provide an overview of hemodynamic changes in cerebral hyperperfusion and endogenous pathways leading to cerebral hyperperfusion in term neonates with HIE.

## Methods

### Study Design

A systematic review was performed following the steps of the Preferred Reporting Items for Systematic Reviews and Meta-Analysis (PRISMA) statement (10). Prior to initiation, the systematic review protocol was registered in PROSPERO (https://www.crd.york.ac.uk/PROSPERO), registration number CRD42020152957. Because the studies in this systematic review were considered to be heterogeneous regarding study population, study technique and study outcome, we refrained from statistically pooling the data in a meta-analysis and performed a best evidence synthesis. The results of this systematic review will be described in a narrative manner.

### Inclusion and Exclusion Criteria

To systematically review the pathophysiology of hyperperfusion in term neonates with hypoxic-ischemic encephalopathy, we conducted a search including papers in which term neonates (>36 weeks' GA) had a diagnosis of HIE caused by perinatal asphyxia, with or without hypothermia treatment. We also included animal studies with models of perinatal HIE at term equivalent age, because pathophysiology is more thoroughly studied in animal models. We excluded reviews, articles on preterm neonates (<36 weeks' GA), articles with pooled data on both term and preterm neonates, articles on adults and articles on neonates with main pathology that alters the brain physiology (main cerebral hemorrhage, metabolic disorders, main cardiac abnormalities or chromosomic disorders). We also excluded articles with data on NIRS or MRI when they did not report effects on vascular resistance, (re)perfusion or cerebral blood volume. No date restrictions were applied. We limited the search to English articles only. When analyzing results, the authors considered rats and mouses as “small animals” and piglets and lambs as “large animals.”

Since autoregulation is not the scope of this review, we did not include articles on this topic specifically. If the reader would like to have an in-depth overview of cerebral vascular autoregulation (CVAR) studies using NIRS in neonates and given that autoregulation is not the main focus of the review, we would like to refer to the paper of Thewissen et al. (11).

### Search Strategy

The research question was defined according to the PICOTS system (Supplementary Appendix 1). The main search terms were Hypoxic-Ischemic Encephalopathy (HIE), Vasodilation and (Re-)perfusion. Besides title and abstract, MeSH-terms were used for all search terms (Supplementary Appendixes 2–4). A medical librarian with systematic review experience helped in developing the search strategy.

### Data Sources, Studies Selections, and Data Extraction

Medline (PubMed) and Embase were searched from inception to December 12, 2018, with similar search strategies. Two researchers (D.K. and K.A.) independently analyzed all abstracts after removing duplicates while appraising the in- and exclusion criteria. The full text of potentially eligible studies was then assessed for inclusion using the same inclusion and exclusion criteria. If an inconsistency occurred during the abstract or full text analysis, a consensus was reached in a meeting or by the involvement of a third researcher (J.D.). Data were extracted and reviewed by D.K. and A.H. The characteristics of the included studies were recorded using data extraction forms, which included the following characteristics: study design, population, number of participants, study technique and relevant findings. Before submission of the review, the search was repeated in Medline and Embase for additional articles.

### Methodological Quality

The Checklist for Cohort Studies from The Joanna Briggs Institute Critical Appraisal Tools was used to assess the methodological quality of the individual studies (12). The methodologic quality was assessed independently by two researchers (D.G.K. and A.H.), and disagreements were resolved in a meeting or by the consideration of a third researcher (J.D). We calculated the score and added up “Yes” as 1; “No” as 0 and “Unclear” as 0. Quality was calculated as the added up score, divided by the number of questions answered with “Yes,” “No” or “Unclear.” (Non-Applicable was not taken into account.) For example, 7 of 11 questions were answered with Yes and 1 question was answered with Non-Applicable. The calculated score is 7/(11–1) = 7/10 = 70%. A score of ≥80% was considered a study of high quality. A score between 60 and 80% was considered as moderate quality, whereas a score of ≤ 60% was considered to be of low quality. These cut-off values were agreed with the research team.

### Best-Evidence Synthesis

A best-evidence synthesis was performed since the outcome measures of the included studies were too heterogeneous for a meta-analysis. We used the guidelines from Proper et al. (13) to synthesize the methodologic quality of the studies and to be able to reach conclusions regarding the pathophysiology of cerebral hyperperfusion. The guideline consists of the following three levels:

1. Strong evidence: consistent findings in multiple (≥2) high-quality studies;
2. Moderate evidence: consistent findings in one high-quality study and at least one low-quality study, or consistent findings in multiple low-quality studies;
3. Insufficient evidence: only one study available or inconsistent findings in multiple (≥2) studies.

Results were considered to be consistent when a minimum of 75% of the studies showed results in the same direction, which was defined according to significance (*p* < 0.05). If there were two or more high-quality studies, the studies of low methodologic quality were disregarded in the evidence synthesis.

## Results

**Figure 2:**
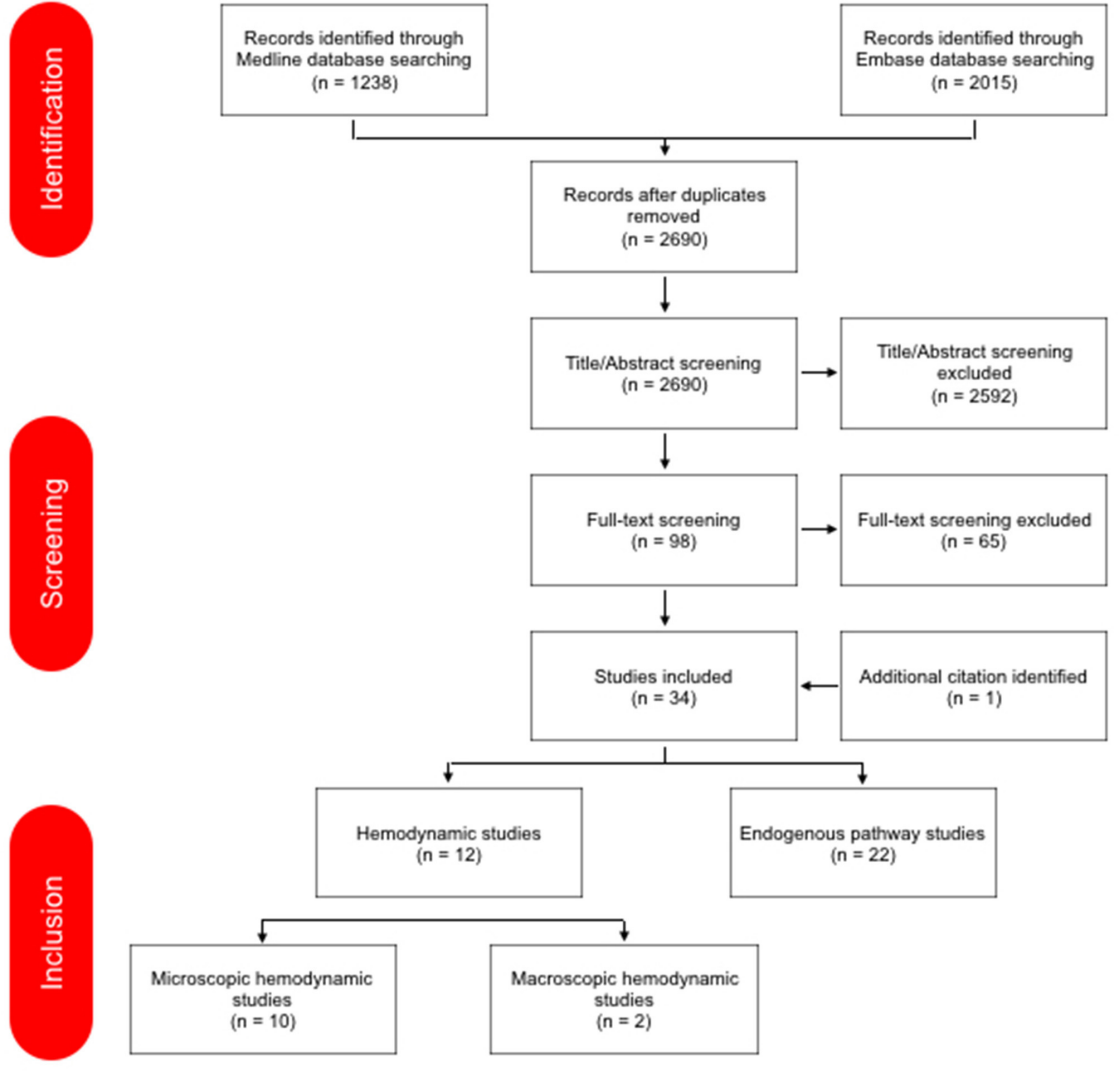
PRISMA flow diagram of the study selection (10).

The search in Medline and Embase provided 3,253 results. Figure 2 provides a detailed overview of study screening and selection. The additional search in Medline and Embase before submission of the review did not provide further articles.

Data from the literature were analyzed and presented below in two main subjects: (1) Hemodynamic Changes subdivided into macro- and microscopic hemodynamic changes, and (2) Endogenous Pathways which was subdivided into NDMA/MAPK, NO, prostanoids, and other endogenous studies. This makes that the review has a total of six themes.

A total of 34 studies were eligible for the review; 12 hemodynamic changes studies and 22 endogenous pathway studies. In total, 44 human newborns and 334 animals (169 large animals; 165 small animals) were included in the hemodynamic changes studies. In the endogenous pathway studies, there were no human newborns studies included. A total of 775 animals (656 large animals; 119 small animals) in the endogenous pathway studies were studied; in five studies (14–18) the number of included animals in the study was unknown. All studies were prospective cohort studies. A separate subject about human studies was not made because there were not enough human studies for that. Characteristics, relevant findings and methodological quality of each study are summarized in Tables 1, 2.

**Table 1:** Study characteristics hemodynamic studies, relevant findings, and methodological quality.

**References**	**Design**	**Population; no**.	**Technique**	**Relevant findings**	**Methodological quality**
**MACROSCOPIC HEMODYNAMIC STUDIES**					
Jinnai et al. (19)	Prospective cohort	Piglets; *N =* 26	Near-infrared TRS	↓CBV within 24 h after the HI-insult, longer duration of LAEEG after insult is associated with a greater decrease in the HT group	High
Wu et al. (20)	Prospective cohort	Human newborns, gestational age 38,8 ± 2 weeks; *N =* 20	Electrical velocimetry, transcranial doppler	During the rewarming phase after HI: ↑CO due to ↑HR; ↑MCA peak systolic value; no changes in CrO_2_ and CFOE, suggesting cerebral flow metabolism coupling remained intact during rewarming	High
Buckley et al. (21)	Prospective cohort	Rat pups; *N =* 46	Diffuse correlation spectroscopy	↑CBF after 5–10 min after the HI-insult in both hemispheres, more pronounced in the contralateral hemisphere	Moderate
Nakamura et al. (22)	Prospective cohort	Piglets; *N =* 21	Near-infrared TRS, Histologic staining	Increased CBV up to 6 h after HI insult indicated more marked histopathological brain damage	Moderate
Wang et al. (23)	Prospective cohort	Rat pups; *N =* 90	Color Doppler Ultrasound, VTQ, HE-staining	After asphyxia, ↑Vd of the MCA, ↓Vs and ↓RI of the MCA after 3 h; consistent with the pathological findings	Low
Chakkarapani et al. (24)	Prospective cohort	Piglets; *N =* 51	aEEG	Cerebrovascular Pressure Reactivity ([PRx; the correlation coefficient between intracranial and mean arterial blood pressure (MABP)]; PRx is impaired during HI, latent phase, secondary PRx peak (after 6 h) is correlated with severe neuropathology	Moderate
Manole et al. (25)	Prospective cohort	Rat pups; *N =* 17	ASL-MRI	After 8,5–9 min of asphyxial CA, subcortical hyperemia at 5 min was followed by a return of CBF to baseline values by 10 min, absence of cortical hyperemia after CA (cortical hypoperfusion)	High
Leffler et al. (26)	Prospective cohort	Piglets; *N =* 24	Radioactively labeled microspheres	Reactive hyperemia throughout the brain (except the cerebrum), peaking by 5 min and subsiding by 20 min of reperfusion	Moderate
Rosenberg et al. (27)	Prospective cohort	Neonatal lambs; *N =* 31	Radioactively labeled microspheres	2–5 h after asphyxia, ↑CBF, cerebral oxygen delivery ↓ and cerebral oxygen consumption remained stable due to a proportional increase in CFOE; impaired cerebral autoregulation. Second finding: postasphyxia cerebral vasodilation is not attributed to the evolution of gross cerebral edema	Moderate
Rosenberg et al. (28)	Prospective cohort	Neonatal lambs; *N =* 9	Radioactively labeled microspheres	↑CBF immediately after asphyxia, cerebral oxygen delivery ↑, cerebral oxygen consumption ↓, CFOE ↓	Moderate
**MICROSCOPIC HEMODYNAMIC STUDIES**					
Shaikh et al. (29)	Prospective cohort	Human newborns (*N =* 24); rat pups (*N =* 12)	ASL-MRI study	↑CBF around day 10 of life and up to 1 month of life; ↑VEGF expression 24-48 h after the HI-event, ↑endothelial cell count 7 and 11 days after the HI-event	High
Domoki et al. (30)	Prospective cohort	Piglets; *N =* 7, 1–2 days old	Closed cranial window, Laser-speckle imaging	Marked cortical hyperemia in 5/7 piglets, ↑pial artery diameters and arterial flow velocity	High

*aEEG, amplitude-integrated electroencephalogram; ASL-MRI, arterial spin-labeled magnetic resonance imaging; CA, cardiac arrest; CBF, cerebral blood flow; CBV, cerebral blood volume; CFOE, cerebral fractional oxygen extraction; CO, cardiac output; CrSO2, regional cerebral oxygen saturation; HE, hematoxylin and eosin; HI, hypoxic ischemic; HT, hypothermia; HR, heart rate; LAEEG, low-amplitude electroencephalography; MCA, middle cerebral artery; MRI, magnetic resonance imaging; TRS, time resolved spectroscopy; Vd, diastolic velocity; VEGF, vascular endothelial growth factor; VTQ, virtual touch tissue quantification*.

**Table 2:** Study characteristics endogenous pathway studies, relevant findings, and methodological quality.

**References**	**Design**	**Population; no**.	**Technique**	**Relevant findings**	**Methodological Quality**
**NMDA/MAPK**					
Dang et al. (31)	Prospective cohort	Piglets; *N =* 25	H-MRS, DWI	The glutamate level in the basal ganglia underwent a “two-phase” change after HI: the first rise in glutamate after 0–6 h and second rise in glutamate after 24–30 h, due to reperfusion injury	High
Armstead et al. (32)	Prospective cohort	Rat pups; *N =* 65	Closed Cranial Window	Treatment with Plasminogen Activator Inhibitor peptide EEIIMD prevents the impairment of vasodilator responses to hypercapnia and hypotension after HI, by upregulating p38 MAPK	High
Kiessling et al. (33)	Prospective cohort	Piglets; *N =* 90	Closed Cranial Window	Inhibition of Urokinase Plasminogen Activator and Integrin prevents impairment of cerebrovasodilation after HI	Moderate
Armstead et al. (34)	Prospective cohort	Rat pups; *N =* 54	Closed Cranial Window	Urokinase Plasminogen Activator impairs cerebrovasodilation through LRP and MAPK	High
Bari et al. (35)	Prospective cohort	Piglets; *N =* 53	Closed Cranial Window	Kynurenine acid (KYNA) attenuates NMDA-induced pial artery dilatation; NMDA-induced arteriolar dilatation can be inhibited by KYNA	High
Philip et al. (36)	Prospective cohort	Newborn lambs; *N =* 42	Closed Cranial Window	Protein thyrosine kinase and MAPK impairs NMDA-induced cerebrovasodilation by nociceptin/orphanin FQ activation	Moderate
Jagolino et al. (37)	Prospective cohort	Newborn lambs; *N =* 119	Closed Cranial Window	Protein thyrosine kinase, MAPK and nociceptin/orphanin FQ impair hypercapnic cerebrovasodilation	Moderate
Perciaccante et al. (38)	Prospective cohort	Piglets; *N =* 60	Intravital microscopy	(1) Hypothermia fails to preserve cerebral arteriolar dilatation to NMDA during and following ischemia; (2) Cerebral vascular responsiveness to an excitatory neurotransmitter is intact despite the reduced metabolic rate during hypothermia	Moderate
Armstead et al. (39)	Prospective cohort	Piglets; *N =* 42	Closed Cranial Window	Nociceptin/Orphalin FQ and NMDA contribute to the impairment of hypotensive cerebrovasodilation	High
Taylor et al. (40)	Prospective cohort	Newborn lambs; *N =* 20	Microinjection into the brain, Doppler imaging	(1) Local microinjection with NMDA increases both local and global CBF within minutes of injection; (2) Most marked increases in the right midbrain, diencephalon and temporal lobe; (3) Alterations in echotexture are primarily due to intracellular cytoplasmic changes and microscopic hemorrhage	High
**NO**					
Hsu et al. (14)	Prospective cohort	Postpartum day-7 rat pups; *N =* ?	Electron microscopy, doppler imaging	Microvascular damage post HI is contributed by neurnal NOS, nNOS underwent a “two-phase” change after HI: first rise in nNOS directly after the HI-event (swollen nucleoli, CBF↓); second rise 3 h after reoxygenation (overactive microglia, ↑CBF)	Moderate
Domoki et al. (41)	Prospective cohort	Piglets; *N =* 45	Closed Cranial Window	NMDA-induced vasodilation is mediated by endothelium-independent nitric oxide release and activation of neuronal NOS positive neurons	Moderate
Dorrepaal et al. (42)	Prospective cohort	Newborn lambs; *N =* 16	unknown	Inhibition of NOS by N-nitro-L-arginine (NLA) restores autoregulation of cerebral bloodflow, suggesting a role for nitric oxide-induced vasodilation in the impairment of autoregulation	Moderate
Wilderman et al. (15)	Prospective cohort	Piglets; *N =* ?	Closed Cranial Window	Neuronally derived NO contributes to hypoxic pial artery dilatation, through the formation of cGMP and the subsequent release of methionine enkephalin and leucine enkephalin	Moderate
Armstead et al. (16)	Prospective cohort	Piglets, *N =* ?	Closed Cranial Window	Contribution of Kca channel activation to hypoxic cerebrovasodilation is not mediated by NO/cGMP	Low
**PROSTANOID**					
Taniguchi et al. (17)	Prospective cohort	Rat pups, cell-specific knockout mouse pups; *N =* ?	unknown	Prostaglandin E2 EP4 receptor is cerebroprotective, it improves cerebral perfusion in both the contralateral and ipsilateral hypoxic-ischemic hemispheres	Moderate
Pourcyrous et al. (43)	Prospective cohort	Piglets; *N =* 15	Radioactive microsphere CBF determination	(1) Brain stem bloodflow increases at 1min of asphyxia, is maintained at 5 min of asphyxia and increases more during reventilation than bloodflow to cerebrum and cerebellum; (2) Inhibition of prostanoid production with indomethacin does not limit vasoconstriction	High
Leffler et al. (26)	Prospective cohort	Piglets; N-12	Radioactive microsphere CBF determination	The failure of hypercapnia to dilate pial arterioles after cerebral ischemia results from the inability of this stimulus to increase cerebral vasodilator prostanoid synthesis	Moderate
Leffler et al. (18)	Prospective cohort	Piglets; *N =* ?	Closed Cranial Window	(1) Prostanoid in cortical subarachnoid CSF increase during acute hypoxia combined with hypercapnia coincident with dilatation of the pial vessels; (2) Systemic indomethacin decreases pial artery dilatation in response to combined hypoxia and hypercapnia	Low
**OTHER**					
Parfenova et al. (44)	Prospective cohort	Piglets; *N =* 26	Intravital microscopy, closed cranial window	CO, produced by astrocytes, has antioxidant effects (HO/CO and CORM-A1) and is cerebroprotective in neonatal asphyxia	High
Wilderman et al. (45)	Prospective cohort	Piglets, *N =* 65	Closed Cranial Window	cAMP contributes to hypoxic pial artery dilatation; endogenous PACAP modulates cAMP-induced opioid release, thereby contributing to hypoxic pial artery dilatation	High
Rosenberg et al. (28)	Prospective cohort	Newborn lambs; *N =* 16	Radioactive microsphere CBF determination	(1) Immediately after 5 min of asphyxia, increased CBF up to 60 min of reperfusion; (2) Damage by oxygen free radicals during postasphyxia cerebral reperfusion is important to the genesis of late postasphyxia bloodflow and oxygen metabolism abnormalities (treatment with activated polyethylene glycol catalase increases the CBF significantly 5 min postasphyxia)	Moderate

*cAMP, cyclic adenosine monophosphate; cGMP, cyclic guanosine monophosphate; CO, carbon monoxide; CORM-A1, CO-releasing molecule-A1; CSF, cerebrospinal fluid; DWI, diffusion-weighted imaging; HI, hypoxia ischemia; H-MRS, proton magnetic resonance imaging; HO, heme oxylase; Kca, calcium sensitive K-channel; LRP, lipoprotein-related protein; MAPK, mitogen-activated protein kinas; NMDA, n-methyl-d-aspartate, NO, nitric oxide, NOS, nitric oxide syntases; PACAP, pituitary adenylate cyclase-activating peptide*.

There were 14 studies of high, 17 studies of moderate and three studies of low methodological quality (Tables 1, 2). Table 3 shows the critical review of the studies, presenting the questions to assess methodological quality and the answers to these questions.

**Table 3:** Methodological analysis of the included studies.

**Study**	**1. Were the two groups similar?**	**2. Were the exposures measured similarly to both groups?**	**3. Was the exposure measured reliably?**	**4. Were confounding factors identified?**	**5. Were strategies to deal with confounding factors stated?**	**6. Were the groups/** **participants free of the outcome at the beginning?**	**7. Were the outcomes measured reliably?**	**8. Was the follow** ** up time** **reported and** **long enough?**	**9. Was** **follow up** **complete?**	**10. Were strategies to address incomplete follow up utilized?**	**11. Was appropriate statistical analysis used?**	
**MACROSCOPIC HEMODYNAMIC STUDIES**												
Jinnai et al. (19)	+	+	+	+	+	+	+	+	+	NA	+	10/10 = 100%
Wu et al. (20)	+	+	+	+	–	–	+	+	+	NA	+	8/10 = 80%
Buckley et al. (21)	+	+	–	+	–	+	+	+	?	–	+	7/11 = 63%
Nakamura et al. (22)	+	+	+	+	–	+	+	–	–	NA	?	6/10 = 60%
Wang et al. (23)	?	+	?	–	NA	+	?	+	–	NA	+	4/9 = 44%
Chakkarapani et al. (24)	+	+	+	–	NA	+	?	–	+	NA	+	6/9 = 67%
Manole et al. (25)	+	+	+	+	–	+	+	–	+	NA	+	8/10 = 80%
Leffler et al. (46)	+	–	–	–	–	+	+	+	+	NA	+	6/10 = 60%
Rosenberg et al. (27)	+	+	+	–	NA	+	+	–	+	NA	+	7/9 = 78%
Rosenberg et al. (28)	+	+	+	–	NA	+	+	–	+	NA	?	6/9 = 67%
**MICROSCOPIC HEMODYNAMIC STUDIES**												
Shaik et al. (29)	+	–	+	+	+	–	+	+	+	NA	+	8/10 = 80%
Domoki et al. (30)	+	+	+	+	–	+	+	+	+	NA	+	9/10 = 90%
**ENDOGENOUS STUDIES—NMDA/MAPK**												
Armstead et al. (32)	+	+	+	+	–	+	+	–	+	NA	+	8/10 = 80%
Kiessling et al. (33)	+	+	+	–	NA	+	+	–	+	NA	+	7/9 = 78%
Armstead et al. (34)	+	+	+	+	–	+	+	–	+	NA	+	8/10 = 80%
Bari et al. (35)	+	+	+	+	–	+	+	–	+	NA	+	8/10 = 80%
Philip et al. (36)	+	+	+	–	NA	+	+	–	+	NA	+	7/9 = 78%
Jagolino et al. (37)	+	–	+	+	–	+	+	?	+	NA	+	7/10 = 70%
Armstead et al. (39)	+	+	+	+	–	+	+	–	+	NA	+	8/10 = 80%
Taylor et al. (40)	+	+	+	+	+	+	+	–	+	NA	+	9/10 = 90%
**ENDOGENOUS STUDIES—NO**												
Hsu et al. (14)	–	?	+	–	NA	+	+	+	+	NA	+	6/9 = 67%
Domoki et al. (41)	+	+	+	–	NA	+	+	?	+	NA	+	7/9 = 78%
Dorrepaal et al. (42)	?	+	+	–	NA	+	+	–	+	NA	+	6/9 = 67%
Wilderman et al. (15)	–	?	+	+	–	+	+	–	+	NA	+	6/10 = 60%
Armstead et al. (16)	–	?	+	–	NA	+	+	–	+	NA	+	5/9 = 56%
**ENDOGENOUS STUDIES—PROSTANOID**												
Taniguchi et al. (17)	–	?	+	+	–	+	+	+	+	NA	+	7/10 = 70%
Pourcyrous et al. (43)	+	+	+	+	+	+	+	–	+	NA	+	9/10 = 90%
Leffler et al. (46)	+	+	+	–	NA	+	+	–	+	NA	?	6/9 = 67%
Leffler et al. (18)	–	?	+	–	NA	+	+	–	+	NA	+	5/9 = 56%
**ENDOGENOUS STUDIES—OTHER**												
Parfenova et al. (44)	+	+	+	+	+	+	+	+	+	NA	+	10/10 = 100%
Dang et al. (31)	+	+	+	+	–	+	–	+	+	NA	+	8/10 = 80%
Perciaccante et al. (38)	?	+	+	+	–	+	+	–	+	NA	+	7/10 = 70%
Wilderman et al. (45)	+	?	+	+	+	+	+	–	+	NA	+	8/10 = 80%
Rosenberg et al. (28)	+	+	+	–	NA	+	+	–	+	NA	+	7/9 = 78%

+ Yes, – No, ? Unclear, NA Not Applicable.

The subsequent following items were assessed: (1). Were the two groups similar and recruited from the same population? (2). Were the exposures measured similarly to assign people to both exposed and unexposed groups? (3). Was the exposure measured in a valid and reliable way? (4). Were confounding factors identified? (5). Were strategies to deal with confounding factors stated? (6). Were the groups/participants free of the outcome at the start of the study (or at the moment of exposure)? (7). Were the outcomes measured in a valid and reliable way? (8). Was the follow up time reported and sufficient to be long enough for outcomes to occur? (9). Was follow up complete, and if not, were the reasons to loss to follow up described and explored? (10). Were strategies to address incomplete follow up utilized? (11). Was appropriate statistical analysis used?

### Macroscopic Hemodynamic Changes

**Figure 3:**
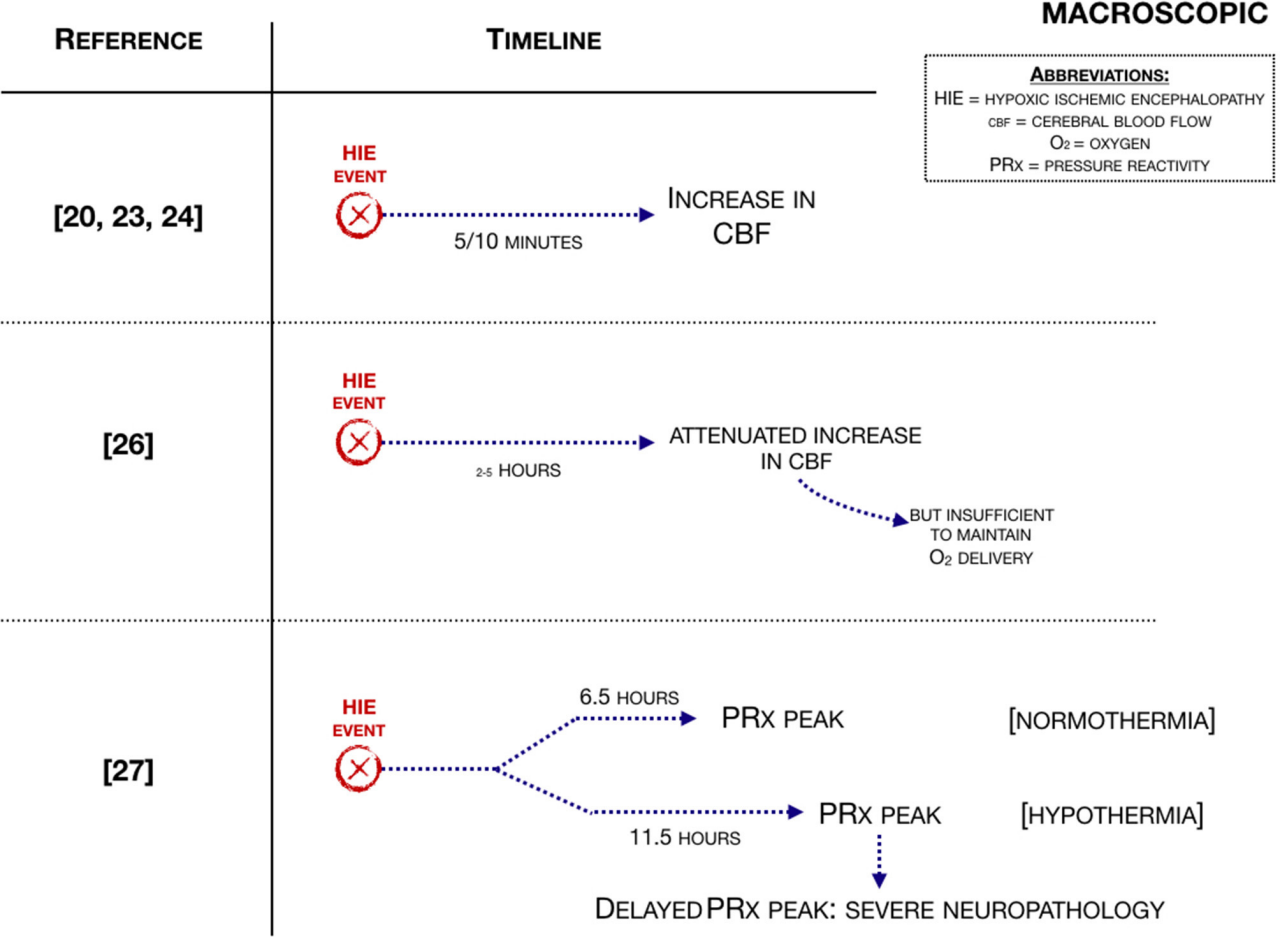
Macroscopic hemodynamic changes associated with an HIE event (21, 24, 25, 27, 28).

Ten studies were included describing hemodynamic changes on a macrovascular level (Figure 3). Regarding small animal studies, Manole et al. (25), showed that after the hypoxic-ischemic (H-I) event cerebral blood flow (CBF) increases within 5 min and subsided after 10 min (studied with MRI). Wang et al. (23) used virtual touch tissue quantification (VTQ), histological staining and ultrasound to determine the severity of brain damage from HIE. In the asphyxia group, the diastolic velocity (Vd) was significantly increased 3 h post carotid ligation compared to the ischemia group, leading to a lower resistance index (RI) of the right MCA, indicating that the elasticity of the cerebral blood vessels decreases. This study suggests that there is a consistent correlation among histological and hemodynamic changes, VTQ values and neurodevelopmental outcome. Additionally, another small animal study showed in what regions hyperperfusion takes place. The study of Buckley et al. (21), showed hyperperfusion in both hemispheres, and more pronounced in the contralateral hemisphere (studied with diffuse correlation spectroscopy).

Moving to large animal studies, Rosenberg studied cerebral blood flow (CBF) in 1986 (28) and in 1988 (27). Rosenberg et al. (27) showed that CBF was increased 2 h after the H-I event, although the increase was attenuated. The same group (28) demonstrated that reactive hyperemia was followed by a period of hypoperfusion. Cerebral oxygen delivery increased, while cerebral oxygen consumption was significantly decreased when compared to control, suggesting mitochondrial dysfunction. Additionally, cerebral fractional oxygen extraction (CFOE) decreased. CBF increased in response to induced hypoxia at 2–5 h after HI, but the increase is attenuated and is insufficient to maintain oxygen delivery. Cerebral oxygen consumption remained stable due to a proportional increase in CFOE (27). Another large animal study showed that an increased CBF subsided after 20 min of reperfusion (26) (studied with radiolabeled microspheres).

The study of Chakkarapani et al. (24) investigated cerebral perfusion by using cerebrovascular pressure reactivity (PRx). In this study, PRx was shown to be impaired during and after a H-I event. The study mentioned that a secondary PRx peak happened after 6.5 h in the normothermia group and after 11.5 h in the hypothermia group and that this was predictive of severe neuropathology and greater insult severity. Nakamura et al. (22), showed that an increased CBF within 6 h after the hypoxic-ischemic event indicates more marked histopathological damage [studied with near-infrared time-resolved spectroscopy (TRS)].

One large animal study investigated in what regions hyperperfusion takes place. Leffler et al. (26), studied hyperperfusion with radioactive-labeled microspheres. This study showed that hyperperfusion was present in the cerebellum, diencephalon, mesencephalon, medulla and spine.

Regarding human newborns, the study of Wu et al. (20) suggested reasons why CBF was increased. Wu et al. (20) used electrical velocimetry and transcranial doppler and indicated that there was an increased cardiac output (CO) in the rewarming phase after hypoxia-ischemia, which was due to an increase in heart rate. This study also showed an increase in the peak systolic velocity of the middle cerebral artery (MCA). The study of Shaikh et al. (29) studied both macroscopic and microscopic hemodynamic changes. This study included term asphyxiated newborns treated with hypothermia. In this study, regional CBF was studied around day 10 and around 1 month of life using MRI and ASL. They found that there was an increased cerebral blood flow around day 10 in life and around 1 month of life, with no reported information between those two points.

### Microscopic Hemodynamic Changes

**Figure 4:**
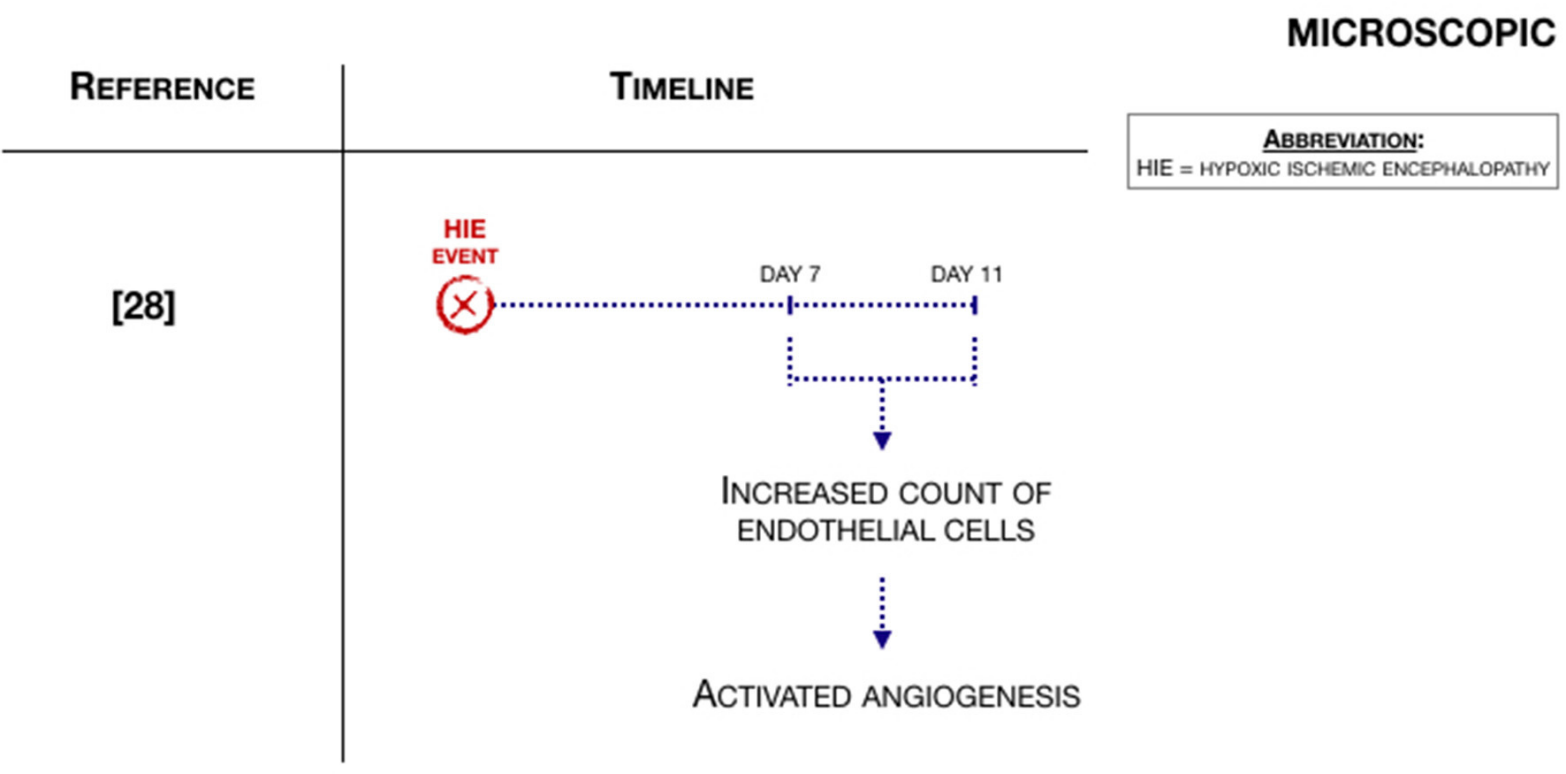
Microscopic hemodynamic changes associated with an HIE event (29).

Two articles also described hemodynamic changes on a microvascular level (29, 30). Shaikh et al. (29), the same study as in the macroscopic hemodynamic changes paragraph, studied angiogenesis in a small animal model of neonatal encephalopathy. They found that Vascular Endothelial Growth Factor (VEGF) was expressed in the first days of life of postmortem human brain tissue and rat pups after the hypoxic-ischemic event. Moreover, the count of endothelial cells was increased on 7 and 11 days after hypoxia-ischemia, indicating that there is active angiogenesis after hypoxia-ischemia (Figure 4).

Regarding large animals, Domoki et al. (30), studied microvascular hemodynamics with laser-speckle imaging. In five out of seven animals, marked cortical hyperemia was found 30 min after the initiation of hypoxia, with an increase in pial arteriolar diameters, simultaneously with arteriolar flow velocity. However, bilateral carotid artery occlusion (BCAO) could not elicit cortical ischemia and the data does not demonstrate a statistically significant effect.

### Endogenous Pathways—NMDA/MAPK

**Figure 5:**
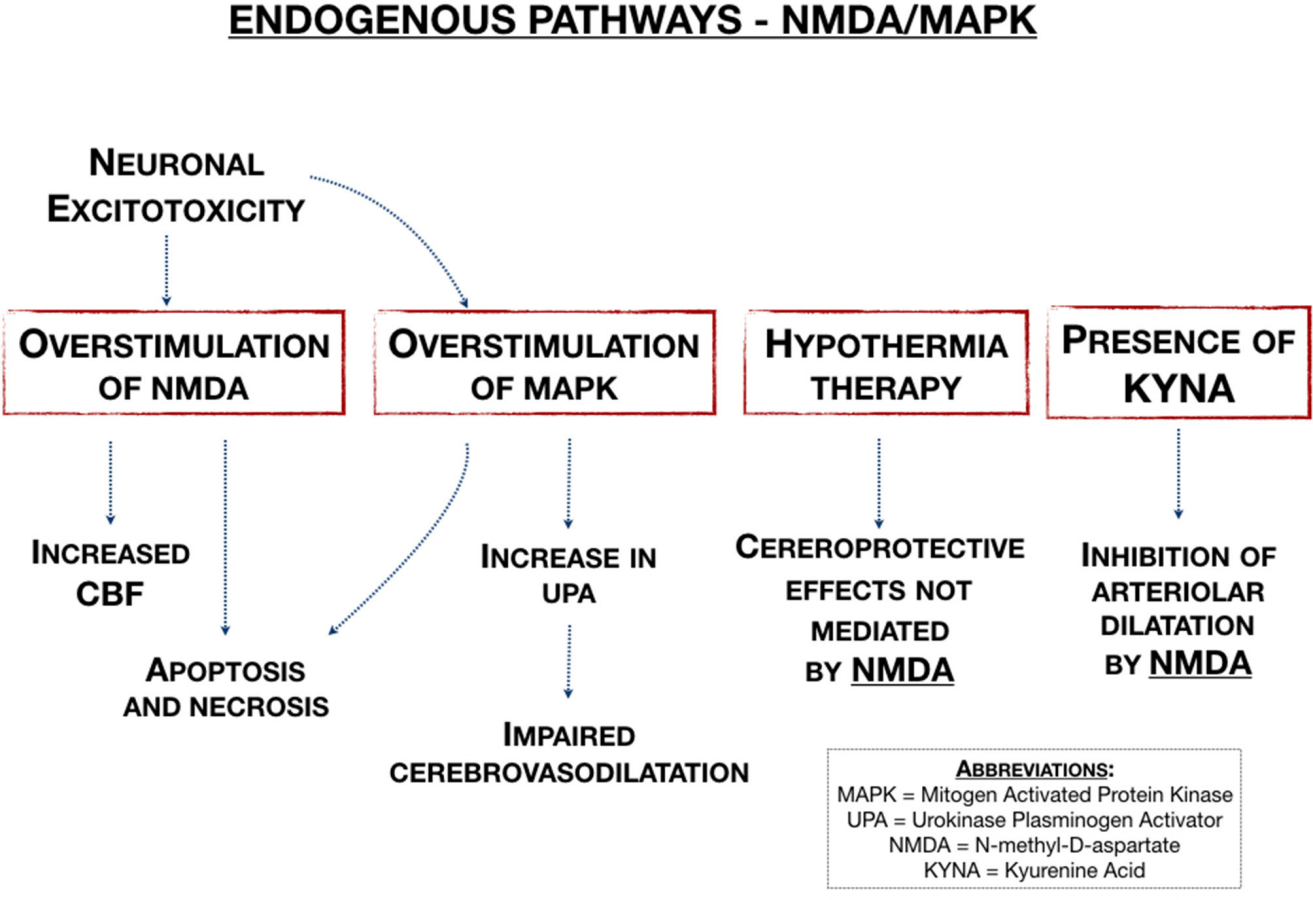
Endogenous pathways associated with NMAD and MAPK (32–35, 38, 40).

In this section, we highlighted studies that described the effects of N-methyl-D-aspartate (NMDA) or mitogen activated protein kinase (MAPK). Figure 5 is a graphical representation of these pathways. The current literature shows that in the process of neuronal excitotoxicity, overstimulation of the NMDA receptor by glutamate triggers the influx of calcium, which leads to apoptosis and necrosis. The MAPK-pathway plays also an important role in neonatal HIE because the overstimulation of this pathway also leads to apoptosis and necrosis. There were three studies (32–34) that all described that Urokinase Plasminogen Activator (uPA) impairs cerebral vasodilation.

Regarding small animal studies, Armstead et al. (34) described that uPA impairs cerebral vasodilation through lipoprotein-related protein (LRP) and the ERK isoform of MAPK. Additionally, Armstead et al. (32) confirms this with a uPA inhibitor and suggests that upregulation of p38 MAPK (a class of MAPK that reacts to stress stimuli) prevents cerebral vasodilation.

Moving to large animal studies, Kiessling et al. (33) described that inhibition of uPA could prevent vasodilatation, suggesting new therapeutic possibilities for clinical practice. On top of that, three other studies (36, 37, 39) described the effects of nociceptin/orphanin FQ and of Protein Tyrosine Kinase, showing that both can partially prevent hypotensive pial dilation impairment, when coadministred with the NMDA antagonist MK801.

The Doppler imaging study of Taylor et al. (40) described that local microinjection with NMDA resulted in increased local and global CBF within minutes of injection. Bari et al. (35) found that NMDA-induced arteriolar dilatation can be inhibited by Kynurenine acid (KYNA). KYNA is a non-competitive antagonist to NMDA receptors and an antagonist to glutamate receptors, suggesting that KYNA attenuates NMDA-induced pial artery dilatation. The study of Perciaccante et al. (38), a study in which the population underwent intravital microscopy, suggests that hypothermia does not affect cerebral arteriolar dilatation to NMDA during and following ischemia, indicating that the cerebroprotective effects of hypothermia therapy are not mediated by NMDA. The study of Dang et al. (31), using proton magnetic resonance spectroscopy (^1^H-MRS) and diffusion-weighted imaging (DWI), showed that the glutamate level in the basal ganglia underwent a “two-phase” change after HI: first a rise in glutamate after 0–6 h and secondly a rise in glutamate after 24–30 h, due to reperfusion injury.

### Endogenous Pathways—NO

**Figure 6:**
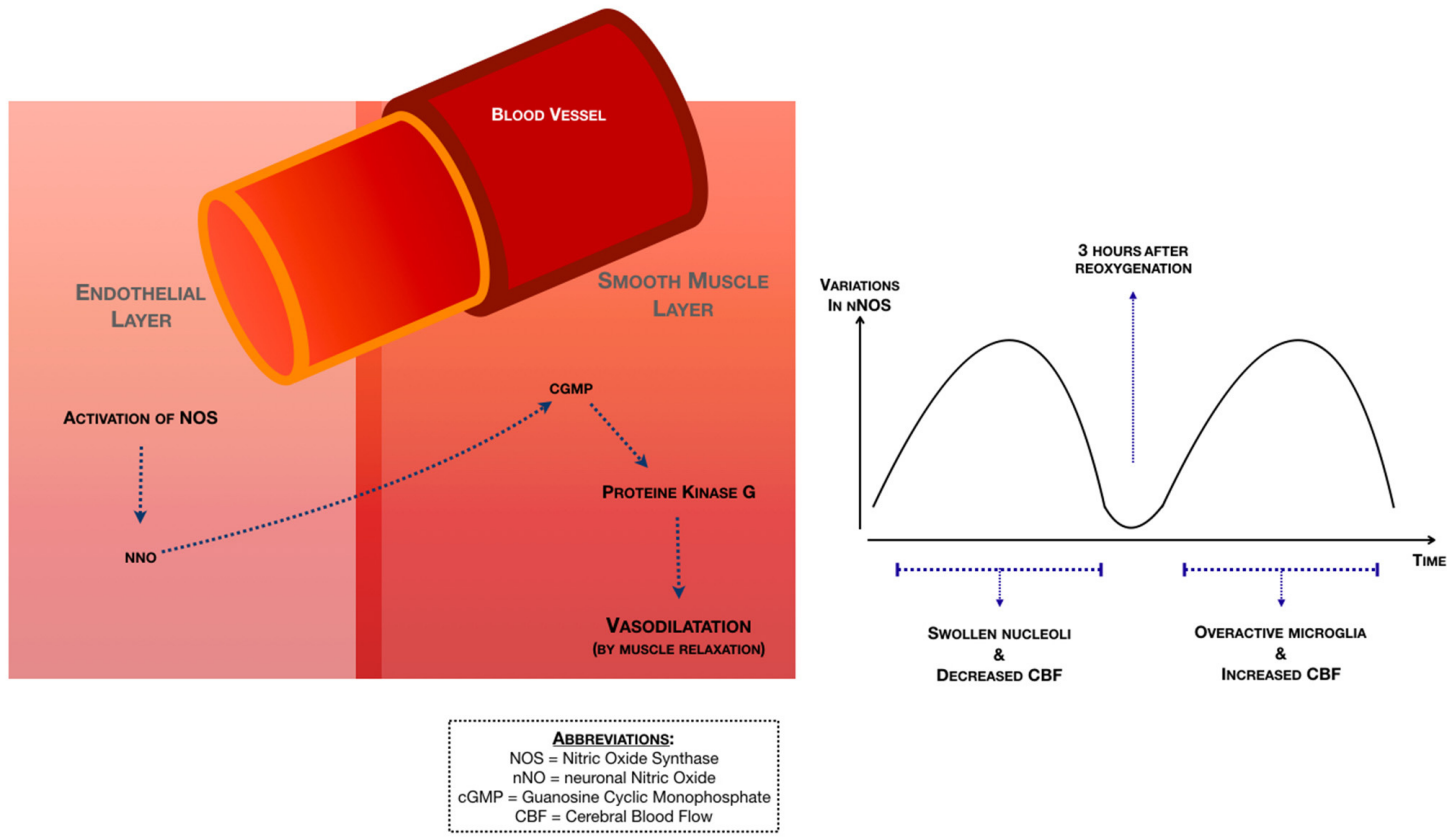
Endogenous pathways associated with NO (14, 41, 42).

Nitric Oxide (NO) plays a major role in vessel dilatation. According to the study of Hsu et al. (14), a study with small animals that underwent Doppler imaging, neuronal NO synthase (nNOS) suffered a “two-phase” change after HI (Figure 6). The first rise in nNOS is directly after the HI-event and is characterized by swollen nuclei and a decreased CBF. The second rise in nNOS happens 3 h after reoxygenation and is characterized by overactive microglia and an increased CBF.

In large animal models, the study of Domoki et al. (41) described the relation between NO and NMDA. This study concluded that NMDA-induced vasodilation is mediated by endothelium-independent NO release and activation of nNOS positive neurons. Dorrepaal et al. (42) showed that neuronally derived NO contributes to hypoxic pial artery dilatation (Figure 6), through the formation of cGMP (cyclic guanosine monophosphate) and the subsequent release of methionine enkephalin and leucine enkephalin. The study of Armstead et al. (16) showed that the contribution of calcium-activated potassium channels to hypoxic cerebral vasodilation is not mediated by NO/cGMP.

### Endogenous Pathways—Prostanoids

**Figure 7:**
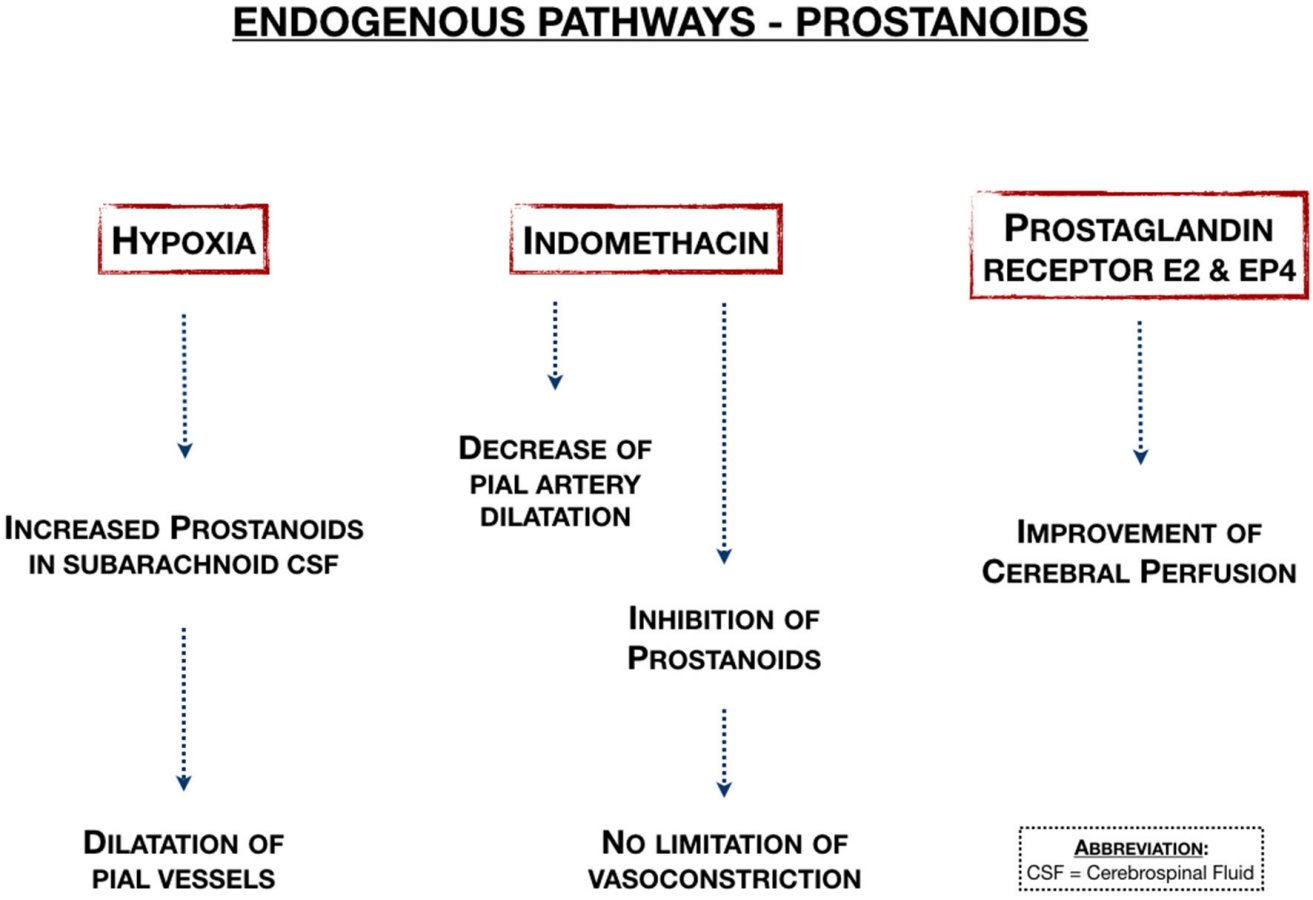
Endogenous pathways associated with prostanoids (17, 18, 43, 46).

Prostanoids may be neuroprotective in neonatal HIE by stimulating the prostanoid E2 receptor (Figure 7). Four studies showed the effects of prostanoids on cerebral blood flow and vasodilation. Taniguchi et al. (17) showed that activation of the prostaglandin E2 EP4 receptor in small animals improved cerebral perfusion in both hemispheres and is therefore neuroprotective. In large animal studies that considered pial vessel mechanisms, Leffler et al. (18) showed that prostanoids were increased in subarachnoid CSF during acute hypoxia combined with hypercapnia, while Pourcyrous et al. (43) showed that inhibition of prostanoid production with indomethacin decreases pial artery dilation in response to combined hypoxia and hypercapnia. In another study from the same group, Leffler et al. (46), they showed that hypercapnia didn't result in dilation of pial arterioles after cerebral ischemia, because of the inability of hypercapnia to increase cerebral vasodilator prostanoid synthesis.

### Endogenous Pathways—Other

Three large animal studies did not fit the two main topics mentioned above. One study that focused on oxygen free radicals: Rosenberg et al. (47) showed that in damage by oxygen free radicals following hypoxia-ischemia, cerebral reperfusion plays a relevant role in the genesis of late postasphyxia blood flow and oxygen metabolism abnormalities. Treatment with polyethylene glycol catalase with oxygen free radicals increases the CBF significantly 5 min after the hypoxic-ischemic event, probably due to increased endothelial permeability. Another study focused on carbon monoxide (44). Carbon monoxide, produced by astrocytes, has antioxidants effects by heme oxylase/ carbon monoxide (HO/CO) and carbon monoxide release molecule-A1 (CORM-A1). HO/CO and CORM-A1 are neuroprotective in perinatal asphyxia, reducing brain oxidative stress and protecting against cerebrovascular dysfunction caused by prolonged neonatal asphyxia. The last study focused on cyclic adenosine monophosphate (cAMP): Wilderman et al. (45) concluded that cAMP contributes to hypoxic pial artery dilatation. Endogenous pituitary adenylate cyclase-activating peptide (PACAP) modulates cAMP-induced opioid release, thereby contributing to hypoxic pial artery dilatation.

### Best-Evidence Synthesis

Overall, we found strong evidence in the studies regarding macroscopic and microscopic hemodynamic changes, the studies regarding NMDA/MAPK and the studies that did not fit the topics and were named in the “other” paragraph. We found moderate evidence in the studies with data on NO and prostanoids. There were no studies that reported insufficient evidence.

There were only two studies that reported data on human neonates (both high-quality studies): Wu et al. (20) and Shaikh et al. (29). Wu et al. (20) mentioned in the macroscopic hemodynamics paragraph, studied 20 human newborns that underwent electrical velocimetry and transcranial doppler. This study indicated that there was an increased cardiac output (CO) in the rewarming phase after hypoxia-ischemia, which was due to an increase in heart rate. This study also showed that the peak systolic velocity of the middle cerebral artery (MCA) was increased. Through NIRS measurements, Wu et al. (20) also mentioned that there were no changes in regional cerebral oxygen saturation (CrO_2_) or cerebral fractional oxygen extraction (CFOE), suggesting that cerebral flow metabolism coupling remained intact during rewarming. Shaikh et al. (29), mentioned in the microscopic hemodynamics paragraph, included 24 term asphyxiated newborns treated with hypothermia. In this study, regional CBF was studied around day 10 and around 1 month of life using MRI and ASL. They found that there was an increased cerebral blood flow around day 10 in life and around 1 month of life, with no reported information between those two points (29).

## Discussion

Cerebral hyperperfusion seen in term neonates with hypoxic-ischemic encephalopathy (HIE) has been correlated with an adverse neurodevelopmental outcome (2, 9, 22–24). In order to understand how cerebral hyperperfusion is correlated with an adverse outcome, we must understand the pathophysiology. We reviewed current literature on the pathophysiology of cerebral hyperperfusion in term neonates with HIE.

Cerebral hyperperfusion may be increased up to 2–5 h after the hypoxic-ischemic event. Moreover, a secondary pressure reactivity peak may be predictive of severe neuropathology. Additionally, therapeutic hypothermia was shown to cause a 5-h delay in cerebral hyperperfusion (11.5 h instead of 6.5 h). Moreover, hyperperfusion is present in the cerebellum, diencephalon, mesencephalon, medulla and spine. Microscopic cerebral hyperperfusion was found to start 10–30 min after the initiation of hypoxia and was still observed around day 10 and around 1 month after birth. Moreover, after the hypoxic-ischemic event, VEGF is expressed and the endothelial cell count is increased, suggesting that angiogenesis is activated after hypoxia-ischemia. On the subject of NMDA and MAPK's endogenous pathway, we found that a rise in uPA can reduce cerebral vasodilation trough MAPK and that Nociceptin/Orphanin FQ contributes to the impairment of cerebral vasodilation. Moreover, NMDA induces vasodilation and hypothermia fails to preserve cerebral dilatation. Due to the reperfusion, there is a second rise in the excitatory amino acid glutamate after 24–30 h and it was also found that NMDA-induced vasodilation is mediated by nNOS through the formation of cGMP. Regarding prostanoids, they were found to be increased during hypoxia-ischemia and their synthesis' inhibition does not limit vasoconstriction. Lastly, prostanoid synthesis may contribute to vasodilation and improve perfusion by the prostaglandin E2 EP4 receptor.

This review suggests that cerebral hyperperfusion may be characterized by angiogenesis and cerebral vasodilation.

Cerebral vasodilation may be mediated by MAPK through uPA, by NMDA through nNOS and by prostanoid synthesis. Inhibition of prostanoid synthesis with indomethacin and inhibition of uPA may limit cerebral vasodilation and therefore may limit cerebral hyperperfusion. By understanding the pathophysiology, we can recognize and comprehend clinical patterns on diagnostic techniques earlier. Early diagnostics of cerebral hyperperfusion in neonates with HIE, can be beneficial for the development of new therapeutics.

There are several diagnostic techniques to detect cerebral hyperperfusion in neonates with HIE. Cranial Ultrasound (cUS) is a powerful and inexpensive alternative tool for MRI. cUS is widely available and can be repeated as often as necessary. cUS has no side effects, and, when performed by an experienced sonographer, provides a wealth of anatomical and functional information. According to Salas et al. (48), characteristic cUS findings of a term neonates with HIE are an increased echogenicity in the thalami, an enhanced gray-white matter differentiation and slit-like ventricles due to edema from the cortical structures. Duplex Ultrasonography (DUS) typically shows decreased resistance index (RI) values (<0.60), as decreased RI values are highly predictive of poor prognosis with either death or severe disability. The study of Archer et al. (49) also described that a very low RI, corresponding to “luxury hyperperfusion,” was correlated with an adverse outcome. One study in our search described a decreased RI of the right MCA in the asphyxia group, compared to the ischemia group (23). This study suggests that with hypoxic-ischemic injury, the elasticity of the brain tissue decreases, and these results are consistent with the pathological findings.

Brain Magnetic Resonance Imaging (MRI) is one of the most used techniques in neonates with HIE and is useful to predict long-term outcomes. In the study of Wintermark et al. (2), 18 asphyxiated neonates underwent MR imaging and ASL-PI (perfusion imaging by arterial spin labeling). In neonates treated with hypothermia, hyperperfusion occurred on day 2–3 in brain areas subsequently exhibiting injury. In neonates with normothermia, hyperperfusion occurred on days 1–6. This study also suggests that early hyperperfusion is correlated with later brain injury even in infants treated with induced hypothermia. Given that this study was not reviewing the pathophysiology but only hemodynamics, we excluded this study in the results. The finding that hypothermia delays the occurrence of hyperperfusion is also found in the study of Chakkarapani et al. (24). In this study, using cerebrovascular pressure reactivity (PRx) in piglets, a secondary PRx peak was associated with severe neuropathology and with greater insult severity and happened after 6.5 h in the normothermia group and after 11.5 h in the hypothermia group.

In the study of De Vis et al. (9), where 28 neonates diagnosed with HIE were assessed using MR imaging, ^1^H-MRS, and ASL MRI, the main finding is that basal ganglia and thalami perfusion is higher in neonates with an adverse outcome. We have not found studies that investigated perfusion in the basal ganglia and the thalami specifically.

### Limitations

There are several limitations in this systematic review. At first, when assessing the methodological quality of the included studies, we made several assumptions: we defined newborn animals as ≤ 7 days old, according to the Medline definition of “perinatal care.” Studies with newborns >7 days old were excluded. When studies did not describe the age of the participated animals, we answered questions 1 and 2 in the critical appraisal as unknown. Additionally, when gender or weight was not described in the study, we assumed that this was a normal distribution of men and female animals and a normal weight. In most of the studies, there was no baseline table for the participating animals, so we assumed that they were healthy newborns without any outcome in the beginning of the study. In agreement with the research team, a follow-up time of ≥24 h was considered to be long enough for cerebral hyperperfusion to occur. Second, because the included studies were too heterogeneous for a meta-analysis, we narratively described the studies and used Proper guidelines for the best-evidence synthesis. For a systematic review, a meta-analysis would be favorable because the precision and accuracy of estimates can be optimized as more data is used, which also means that it may increase the statistical power to detect an effect. Only a few studies in our search were studies on human neonates. The aim of this review is to give an overview of the knowledge so far and make way for new research ideas, which can come from both human and animal populations. The fact that some of the studies considered in this review were related to animal models, means that age-estimation and correlations to human newborn models may pose as a challenge for future research. Moreover, one must consider the fact that small and large animals have different pathophysiologic mechanisms, and that should be taken into account when considering the different results. Finally, in five studies (14–18) the number of animals participated in the study is unknown, therefore generalization to other animal studies was impossible. Because of various limitations, the results have to be treated with caution.

## Conclusion

The main findings are that cerebral hyperperfusion is present 10–30 min after the hypoxic-ischemic event and persisted around day 10 and up to 1 month of life. Cerebral hyperperfusion may be characterized by angiogenesis and cerebral vasodilation. We found that a rise in prostanoids can reduce cerebral vasodilation trough MAPK and that Nociceptin/Orphanin FQ contributes to the impairment of cerebral vasodilation. Moreover, NMDA induces vasodilation and the cerebroprotective effects of hypothermia therapy are not mediated by NMDA. Due to reperfusion, there is a second rise in the excitatory amino acid glutamate after 24–30 h and it was also found that NMDA-induced vasodilation is mediated by nNOS through the formation of cGMP. Regarding prostanoids, they were found to be increased during hypoxia-ischemia and their synthesis' inhibition does not limit vasoconstriction. Lastly, prostanoid synthesis may contribute to vasodilation and improve perfusion by the prostaglandin E2 EP4 receptor.

### Implications and Suggestions for Future Research

The exact mechanism of cerebral hyperperfusion is not known yet, but we showed why it is clinically important. To develop new therapeutics for neonatal HIE, future research about the role of NMDA and MAPK and the implications of agents that can inhibit uPA or prostanoids, such as indomethacin in cerebral vasodilation needs to be developed. Given that there were only three studies (24, 29, 38) that described the effect of hypothermia therapy on cerebral hyperperfusion, evidence is limited and therefore, future research should also focus on this. Moreover, further research is required to translate these findings into clinical practice. These findings should be taken into account simultaneously with brain imagining techniques, as they present themselves as a valuable asset in assessing the neurodevelopment throughout days/weeks after the hypoxic-ischemic event.

## Data Availability Statement

The original contributions presented in the study are included in the article/Supplementary Material, further inquiries can be directed to the corresponding authors.

## Author Contributions

DK contributed to the study concept, screened titles and abstracts, analyzed the data, and wrote the article. FC prepared the figures and contributed with writing the article. KA screened titles and abstracts and analyzed the data. AH, TA, and FG critically reviewed the article. MB and JD directed the project. All authors contributed to the article and approved the submitted version.

## Conflict of Interest

The authors declare that the research was conducted in the absence of any commercial or financial relationships that could be construed as a potential conflict of interest.

## Supplementary Material

Supplementary Appendix 1 Review question defined according to the PICOTS system.

Supplementary Appendix 2 Summarized search strategy.

Supplementary Appendix 3 Medline search strategy.

Supplementary Appendix 4 Medline search string.

## Abbreviations

AMPA: 2-(aminomethyl)phenylacetic acid
LRP: Lipoprotein-Related Protein
ASL: Arterial Spin Labeling
MABP: Mean Arterial Blood Pressure
BCAO: Bilateral Carotid Artery Occlusion
MAPK: Mitogen activated protein kinase
cAMP: Cyclic Adenosine Monophosphate
MCA: Middle Cerebral Artery
CBF: Cerebral Blood Flow
MRI: Magnetic Resonance Imaging
CFOE: Cerebral Fractional Oxygen Extraction
NIRS: Near-Infrared Spectroscopy
cGMP: Guanosine 3',5'-cyclic Monophosphate
NMDA: N-methyl-D-aspartate
CO: Cardiac Output
(n)NO: (neuronal) Nitric Oxide
CORM-A1: Carbon Monoxide Release Molecule-A1
PACAP: Pituitary Adenylate Cyclase-Activating Peptide
CrO2: Cerebral Oxygen Saturation
PRISMA: Preferred Reporting Items for Systematic Reviews and Meta-Analysis
CSF: Cerebral-Spinal Fluid
PRx: Pressure Reactivity
DWI: Diffusion-Weighted Imaging
RI: Resistance Index
GA: Gestational Age
uPA: Urokinase Plasminogen Activator
HIE: Hypoxic-Ischemic Encephalopathy
US: Ultrasound
HO/CO: Heme Oxylase/Carbon Monoxide
V_d_: Diastolic Velocity
KA: Kainite
VEGF: Vascular Endothelial Growth Factor
KYNA: Kynurenine Acid
VTQ: Virtual Touch Quantification.
