# Supplementary material for: Pathophysiology of Cerebral Hyperperfusion in Term Neonates With Hypoxic-Ischemic Encephalopathy: A Systematic Review for Future Research
Source: Front Pediatr. 2021 Feb 2;9:631258. doi: 10.3389/fped.2021.631258 (PMC7884860; doi:10.3389/fped.2021.631258)
Supplement: Supplementary Appendix 1 — Review question defined according to the PICOTS system. [file Data_Sheet_1.docx]

| **PICOTS** | **Explanation** |
| --- | --- |
| P | Newborn term neonates (>36 weeks’ GA) with a diagnosis of HIE caused by perinatal asphyxia, with or without hypothermia treatment. We also included animal studies with models of perinatal HIE at term equivalent age. We defined newborn animals as ≤ 7 days old. |
| I | No specific intervention |
| C | No specific comparison |
| O | Pathophysiology of cerebral hyperperfusion |
| T | A follow-up time of ≥ 24 hours was considered to be long enough for cerebral hyperperfusion to occur. |
| S | For animals no specific setting, for humans in the neonatal intensive care unit (NICU). |

**Appendix S1**: Review question defined according to the PICOTS system.

| **MeSH** | **TiAb** |
| --- | --- |
| Hypoxia-Ischemia, brain | Hypoxia-Ischemia, brain |
|  | Hypoxic ischaemic encephalopathies |
|  | Hypoxic ischaemic encephalopathy |
|  | HIE |
| Asphyxia Neonatorum | Asphyxia Neonatorum |
|  | Neonatal asphyxia |
|  | Perinatal asphyxia |
| Asphyxia | Asphyxia |
| Hypoxia, brain | Hypoxia, brain |
| Brain ischemia | Brain ischemia |
|  | Hypoxic ischemia |
|  | Cerebral hypoxia ischemia |
|  | Ischemic brain injury |
|  | Hypoxic ischemic injury |
|  | Hypoxic brain injury |
|  | HII |
| AND |  |
| Infant, Newborn | Infant, Newborn |
| Infant | Infant |
|  | Neonat* |
|  | Infant* |
|  | Newborn* |
|  | Antenat* |
|  | Perinat* |
|  | Postnat* |
| AND |  |
| Vasodilation | Vasodilat* |
|  | Luxury perfusion |
|  | Hyperperfusion |
|  | Brain perfusion |
| Perfusion | Perfusion |
| Reperfusion | Reperfusion |
| Spectroscopy, near-infrared | Spectroscopy, near-infrared |
|  | NIRS |
| Ultrasonography, doppler, transcranial | Ultrasonography, doppler, transcranial |
| Ultrasonography, doppler, color | Ultrasonography, doppler, color |
|  | Transcranial doppler ultrasonography |
|  | Color doppler ultrasonography |
|  | Transcranial color doppler ultrasonography |
|  | Transcranial doppler |
|  | TCD |
|  | TCCD |
|  | Cerebral doppler |
|  | Cerebral color doppler |
|  | Color doppler |
|  | Susceptibility weighted imaging |
|  | SWI |
|  | Arterial spin labeling |
|  | ASL |
|  | Q-flow |
| * Truncation symbol was used to find terms with other endings or an alternative spelling. | |

**Appendix S2**: Summarized search strategy.

1. hypoxia-ischemia, brain [MeSH Terms]
2. hypoxia-ischemia, brain [Title/Abstract]
3. hypoxic ischaemic encephalopathies [Title/Abstract]
4. hypoxic ischaemic encephalopathy [Title/Abstract]
5. ischaemic hypoxic encephalopathies [Title/Abstract]
6. ischaemic hypoxic encephalopathy [Title/Abstract]
7. HIE [Title/Abstract]
8. hypoxic-ischaemic encephalopathies [Title/Abstract]
9. hypoxic-ischaemic encephalopathy [Title/Abstract]
10. ischaemic-hypoxic encephalopathies [Title/Abstract]
11. ischaemic-hypoxic encephalopathy [Title/Abstract]
12. 1 – 11 [OR]
13. asphyxia neonatorum [MeSH Terms]
14. asphyxia neonatorum [Title/Abstract]
15. neonatal asphyxia [Title/Abstract]
16. asphyxia neonatal [Title/Abstract]
17. perinatal asphyxia [Title/Abstract]
18. asphyxia perinatal [Title/Abstract]
19. asphyxia [MeSH Terms]
20. asphyxia [Title/Abstract]
21. 13 – 20 [OR]
22. ischemic anoxic encephalopathies [Title/Abstract]
23. anoxic ischemic encephalopathies [Title/Abstract]
24. ischemic anoxic encephalopathy [Title/Abstract]
25. anoxic ischemic encephalopathy [Title/Abstract]
26. ischaemic anoxic encephalopathies [Title/Abstract]
27. anoxic ischaemic encephalopathies [Title/Abstract]
28. ischaemic anoxic encephalopathy [Title/Abstract]
29. anoxic ischaemic encephalopathy [Title/Abstract]
30. 22 – 29 [OR]
31. hypoxia, brain [MeSH Terms]
32. hypoxia, brain [Title/Abstract]
33. brain ischemia [MeSH Terms]
34. brain ischemia [Title/Abstract]
35. hypoxic ischemia [Title/Abstract]
36. hypoxic ischaemia [Title/Abstract]
37. cerebral hypoxia ischaemia [Title/Abstract]
38. cerebral ischemia hypoxia [Title/Abstract]
39. cerebral ischaemia hypoxia [Title/Abstract]
40. brain hypoxia ischaemia [Title/Abstract]
41. brain ischaemia hypoxia [Title/Abstract]
42. brain ischaemia [Title/Abstract]
43. cerebral ischaemia [Title/Abstract]
44. 31 – 43 [OR]
45. ischemic brain injury [Title/Abstract]
46. ischaemic brain injury [Title/Abstract]
47. asphyxial [Title/Abstract]
48. asphyxiated [Title/Abstract]
49. hypoxic ischemic injury [Title/Abstract]
50. hypoxic ischaemic injury [Title/Abstract]
51. hypoxic-ischemic injury [Title/Abstract]
52. hypoxic-ischaemic injury [Title/Abstract]
53. hypoxic brain injury [Title/Abstract]
54. HII [Title/Abstract]
55. 45 – 54 [OR]
56. 12 OR 21 OR 30 OR 44 OR 55
57. Infant, Newborn [MeSH Terms]
58. Infant, Newborn [Title/Abstract]
59. Infant [MeSH Terms]
60. Infant [Title/Abstract]
61. Neonat* [Title/Abstract]
62. Infant* [Title/Abstract]
63. Newborn* [Title/Abstract]
64. Antenat* [Title/Abstract]
65. Perinat* [Title/Abstract]
66. Postnat* [Title/Abstract]
67. 57 – 66 [OR]
68. Vasodilation [MeSH Terms]
69. vasodilat* [Title/Abstract]
70. luxury perfusion [Title/Abstract]
71. hyperperfusion [Title/Abstract]
72. hyper perfusion [Title/Abstract]
73. brain perfusion [Title/Abstract]
74. perfusion [MeSH Terms]
75. perfusion [Title/Abstract]
76. reperfusion [Title/Abstract]
77. reperfusion [MeSH Terms]
78. 68 – 77 [OR]
79. Ultrasonography, doppler, transcranial [MeSH Terms]
80. Ultrasonography, doppler, color [MeSH Terms]
81. transcranial doppler ultrasonography [Title/Abstract]
82. color doppler ultrasonography [Title/Abstract]
83. transcranial color doppler ultrasonography [Title/Abstract]
84. transcranial colour doppler ultrasonography [Title/Abstract]
85. trans cranial color doppler ultrasonography [Title/Abstract]
86. trans cranial colour doppler ultrasonography [Title/Abstract]
87. transcranial doppler [Title/Abstract]
88. trans cranial doppler [Title/Abstract]
89. TCD [Title/Abstract]
90. transcranial color doppler [Title/Abstract]
91. trans cranial color doppler [Title/Abstract]
92. transcranial colour doppler [Title/Abstract]
93. trans cranial colour doppler [Title/Abstract]
94. TCCD [Title/Abstract]
95. erebral doppler [Title/Abstract]
96. cerebral color doppler [Title/Abstract]
97. cerebral colour doppler [Title/Abstract]
98. colour doppler [Title/Abstract]
99. color doppler [Title/Abstract]
100. 79 – 99 [OR]
101. susceptibility weighted imaging [Title/Abstract]
102. SWI [Title/Abstract]
103. 101 OR 102
104. arterial spin labeling [Title/Abstract]
105. ASL [Title/Abstract]
106. arterial spin labelling [Title/Abstract]
107. 104 – 106 [OR]
108. Q-flow [Title/Abstract]
109. Q flow [Title/Abstract]
110. 108 OR 109
111. 78 OR 100 OR 103 OR 107 OR 110
112. 56 AND 67 AND 111

**Appendix S3**: Medline search strategy in steps.

((((((((((hypoxia-ischemia, brain [MeSH Terms] OR hypoxia-ischemia, brain [Title/Abstract] OR hypoxic ischaemic encephalopathies [Title/Abstract] OR hypoxic ischaemic encephalopathy [Title/Abstract] OR ischaemic hypoxic encephalopathies [Title/Abstract] OR ischaemic hypoxic encephalopathy [Title/Abstract] OR HIE [Title/Abstract])) OR (hypoxic-ischaemic encephalopathies [Title/Abstract] OR hypoxic-ischaemic encephalopathy [Title/Abstract] OR ischaemic-hypoxic encephalopathies [Title/Abstract] OR ischaemic-hypoxic encephalopathy [Title/Abstract])) OR (asphyxia neonatorum [MeSH Terms] OR asphyxia neonatorum [Title/Abstract] OR neonatal asphyxia [Title/Abstract] OR asphyxia neonatal [Title/Abstract] OR perinatal asphyxia [Title/Abstract] OR asphyxia perinatal [Title/Abstract] OR asphyxia [MeSH Terms] OR asphyxia [Title/Abstract])) OR (ischemic anoxic encephalopathies [Title/Abstract] OR anoxic ischemic encephalopathies [Title/Abstract] OR ischemic anoxic encephalopathy [Title/Abstract] OR anoxic ischemic encephalopathy [Title/Abstract] OR ischaemic anoxic encephalopathies [Title/Abstract] OR anoxic ischaemic encephalopathies [Title/Abstract] OR ischaemic anoxic encephalopathy [Title/Abstract] OR anoxic ischaemic encephalopathy [Title/Abstract])) OR (hypoxia, brain [MeSH Terms] OR hypoxia, brain [Title/Abstract] OR brain ischemia [MeSH Terms] OR brain ischemia [Title/Abstract] OR hypoxic ischemia [Title/Abstract] OR hypoxic ischaemia [Title/Abstract] OR cerebral hypoxia ischaemia [Title/Abstract] OR cerebral ischemia hypoxia [Title/Abstract] OR cerebral ischaemia hypoxia [Title/Abstract] OR brain hypoxia ischaemia [Title/Abstract] OR brain ischaemia hypoxia [Title/Abstract] OR brain ischaemia [Title/Abstract] OR cerebral ischaemia [Title/Abstract])) OR (ischemic brain injury [Title/Abstract] OR ischaemic brain injury [Title/Abstract] OR asphyxial [Title/Abstract] OR asphyxiated [Title/Abstract] OR hypoxic ischemic injury [Title/Abstract] OR hypoxic ischaemic injury [Title/Abstract] OR hypoxic-ischemic injury [Title/Abstract] OR hypoxic-ischaemic injury [Title/Abstract] OR hypoxic brain injury [Title/Abstract] OR HII [Title/Abstract]))) AND ((Infant, Newborn [MeSH Terms] OR Infant, Newborn [Title/Abstract] OR Infant [MeSH Terms] OR Infant [Title/Abstract] OR Neonat* [Title/Abstract] OR Infant* [Title/Abstract] OR Newborn* [Title/Abstract] OR Antenat* [Title/Abstract] OR Perinat* [Title/Abstract] OR Postnat* [Title/Abstract]))) AND (((((((Vasodilation [MeSH Terms] OR vasodilat* [Title/Abstract] OR luxury perfusion [Title/Abstract] OR hyperperfusion [Title/Abstract] OR hyper perfusion [Title/Abstract] OR brain perfusion [Title/Abstract] OR perfusion [MeSH Terms] OR perfusion [Title/Abstract] OR reperfusion [Title/Abstract] OR reperfusion [MeSH Terms])) OR (Spectroscopy, near-infrared [MeSH Terms] OR Spectroscopy, near-infrared [Title/Abstract] OR NIRS [Title/Abstract])) OR (Ultrasonography, doppler, transcranial [MeSH Terms] OR Ultrasonography, doppler, color [MeSH Terms] OR transcranial doppler ultrasonography [Title/Abstract] OR color doppler ultrasonography [Title/Abstract] OR transcranial color doppler ultrasonography [Title/Abstract] OR transcranial colour doppler ultrasonography [Title/Abstract] OR trans cranial color doppler ultrasonography [Title/Abstract] OR trans cranial colour doppler ultrasonography [Title/Abstract] OR transcranial doppler [Title/Abstract] OR trans cranial doppler [Title/Abstract] OR TCD [Title/Abstract] OR transcranial color doppler [Title/Abstract] OR trans cranial color doppler [Title/Abstract] OR transcranial colour doppler [Title/Abstract] OR trans cranial colour doppler [Title/Abstract] OR TCCD [Title/Abstract] OR cerebral doppler [Title/Abstract] OR cerebral color doppler [Title/Abstract] OR cerebral colour doppler [Title/Abstract] OR colour doppler [Title/Abstract] OR color doppler [Title/Abstract])) OR (susceptibility weighted imaging [Title/Abstract] OR SWI [Title/Abstract])) OR (arterial spin labeling [Title/Abstract] OR ASL [Title/Abstract] OR arterial spin labelling [Title/Abstract])) OR (Q-flow [Title/Abstract] OR Q flow [Title/Abstract])))

**Appendix S4**: Medline search string.
